# An orally available Mpro/TMPRSS2 bispecific inhibitor with potent anti-coronavirus efficacy in vivo

**DOI:** 10.21203/rs.3.rs-5454588/v1

**Published:** 2024-11-21

**Authors:** Hin Chu, Huiping Shuai, Jingxin Qiao, Chaemin Yoon, Guo Zhang, Yuxin Hou, Xiaoyan Xia, Lei Wang, Xinyue Deng, Yifei Wang, Qingquan Li, Lianzhao Du, Yuanchen Liu, Minmin Zhou, Hoi Ting Wong, Huan Liu, Bingjie Hu, Yan Chen, Zhen Fang, Ziyi Xia, Yue Chai, Jialu Shi, Yang Wang, Tianrenzheng Zhu, Honglei Zhang, Shuofeng Yuan, Jie Zhou, Jasper Chan, Kwok-Yung Yuen, Chunfu Xu, Jian Lei, Shengyong Yang

**Affiliations:** The University of Hong Kong; The University of Hong Kong; Sichuan University; The University of Hong Kong; Sichuan University; The University of Hong Kong; Sichuan University; The University of Hong Kong; Sichuan University; Sichuan University; National Institute of Biological Sciences; The University of Hong Kong; The University of Hong Kong; The University of Hong Kong; The University of Hong Kong; The University of Hong Kong; The University of Hong Kong; Sichuan University; West China Hospital, Sichuan University; Sichuan University; The University of Hong Kong; The University of Hong Kong; The University of Hong; The University of Hong Kong; The University of Hong Kong; The University of Hong Kong; the University of Hong Kong; The University of Hong Kong; The University of Hong Kong; National Institute of Biological Sciences, Beijing; Sichuan University; Sichuan University

**Keywords:** Mpro, TMPRSS2, SARS-CoV-2, bispecific inhibitor, in vivo, COVID-19

## Abstract

Coronaviruses have caused three major endemics in the past two decades. Alarmingly, recent identification of novel zoonotic coronaviruses that caused human infections suggests the risk of future coronavirus outbreak caused by spillover infection from animal reservoirs remains high^1,2^. Therefore, development of novel therapeutic options with broad-spectrum anti-coronavirus activities are urgently needed. Here, we develop an orally-available bispecific inhibitor, TMP1, which simultaneously targets key coronavirus replication protease M^pro^ and the essential airway protease TMPRSS2^3,4^. TMP1 shows broad-spectrum protection not only against different SARS-CoV-2 variants but also against multiple human-pathogenic coronaviruses in vitro. By using the K18-hACE2 transgenic mouse, hDPP4 knock-in mouse and golden Syrian hamster models, we demonstrate TMP1 cross-protects against highly-pathogenic coronaviruses (SARS-CoV-1, SARS-CoV-2 and MERS-CoV) in vivo and efficiently abrogates SARS-CoV-2 transmission. Through structural and mutagenesis studies, we confirmed the direct interaction of TMP1 with M^pro^ and TMPRSS2, and pinpoint the key sites of interactions. Importantly, TMP1 inhibits the infection of nirmatrelvir-resistant SARS-CoV-2 escape mutants. Together, our findings demonstrate the antiviral potential of the novel bispecific M^pro^/TMPRSS2 antiviral design against human-pathogenic coronaviruses and other emerging coronaviruses.

Coronavirus infections have long been prevalent in the human population. Apart from the four endemic human coronaviruses (HCoV-229E, -NL63, -OC43 and -HKU1)^5^, the highly pathogenic severe acute respiratory syndrome coronavirus (SARS-CoV-1) and Middle East respiratory syndrome coronavirus (MERS-CoV) have caused major outbreaks manifested with severe viral pneumonia^6,7^. In 2019, severe acute respiratory syndrome coronavirus 2 (SARS-CoV-2) emerged and have resulted in the global coronavirus disease 2019 (COVID-19) pandemic, which has accounted for over seven million deaths worldwide to date^8,9^. Moreover, recent identification of the porcine deltacoronavirus (Hu-PDCoV) and canine coronavirus human pneumonia 2018 (CCoV-HuPn-2018)^1,2^ alarmingly underscored the increasing possibility of coronavirus spillover to infect humans. Given the current situation, the chance of future emergence of zoonotic coronaviruses remains high. To prepare for the emerging coronavirus outbreaks, development of effective therapeutic options with pan-coronavirus efficacy are urgently needed.

Coronavirus entry mediated by the spike (S) protein is the first step to establish successful virus infection. To mediate efficient coronavirus entry, sequential cleavage of coronavirus S protein at the S1/S2 and the S2’ site by host proteases is essential. Although recent evidence suggested that a variety of transmembrane proteases could facilitate S protein cleavage^10,11^, TMPRSS2 is predominantly used by coronaviruses to mediate virus entry at the plasma membrane^12–16^. Alternatively, coronavirus entry can take place in the endosomes that requires cathepsin B/L^17^. A wealth of studies has therefore developed anti-coronavirus host-directed antivirals (HDAs) based on these important host proteases^17–19^. Blockade of the endosomal entry pathway sufficiently aborted coronavirus infection in cells with little to no TMPRSS2 expression ^17,20^. Yet in the airway epithelium where abundant TMPRSS2 is expressed^16,21^, TMPRSS2-dependent virus entry remains as the dominant pathway utilized by human-pathogenic coronaviruses^3,12–16,22,23^. In addition, TMPRSS2 is a key determinant that impacts coronavirus transmission and virus-induced tissue pathologies in the infected host^24–26^, suggesting the indispensable role of TMPRSS2 to coronavirus entry and pathogenesis at the primary infection sites.

After coronavirus entry into the host cell, its positive-sense RNA genome is translated into two long two viral polypeptides pp1a and pp1ab. The viral main protease (M^pro^) is a cysteine protease that responsible for the release of 12 out the 16 replicases vital to virus replication by enzymatic cleavage of pp1a and pp1ab^27,28^. Resolution of the M^pro^ crystal structures of coronaviruses demonstrated that their 3D structures were highly conserved^29^. Interestingly, M^pro^ specifically recognizes a glutamine residue at the P1 position of the substrate, which is a unique feature that is not shared by any of the host cysteine protease^4,30^. Therefore, M^pro^ has been an attractive antiviral target for the development of anti-coronavirus therapy^31–34^.

Here, we describe an orally-available M^pro^/TMPRSS2 bispecific inhibitor, TMP1, which simultaneously targets coronavirus entry and replication with potent pan-coronavirus antiviral efficacy both in vitro and in vivo. Notably, we show that TMP1 prevents severe infection in the lower respiratory tract, rescues lethal SARS-CoV-2 infection, and blocks virus transmission. Furthermore, we reveal a differential mode of action of TMP1 when compared to nirmatrelvir, which effectively protects the host from infection of nirmatrelvir-resistant SARS-CoV-2 mutants. In keeping with its activity against SARS-CoV-2, TMP1 similarly attenuates the replication of SARS-CoV-1 and MERS-CoV in animals. Taken together, we have proposed a unique antiviral design of simultaneously targeting the most essential viral and host protease to achieve potent antiviral protection. The development of bispecific antivirals with broad-spectrum efficacy can increase our preparedness against the next coronavirus pandemics.

## Results

### Discovery of M^pro^/TMPRSS2 bispecific inhibitor with highly potent anti-coronavirus efficacy in vitro

**Figure 1:**
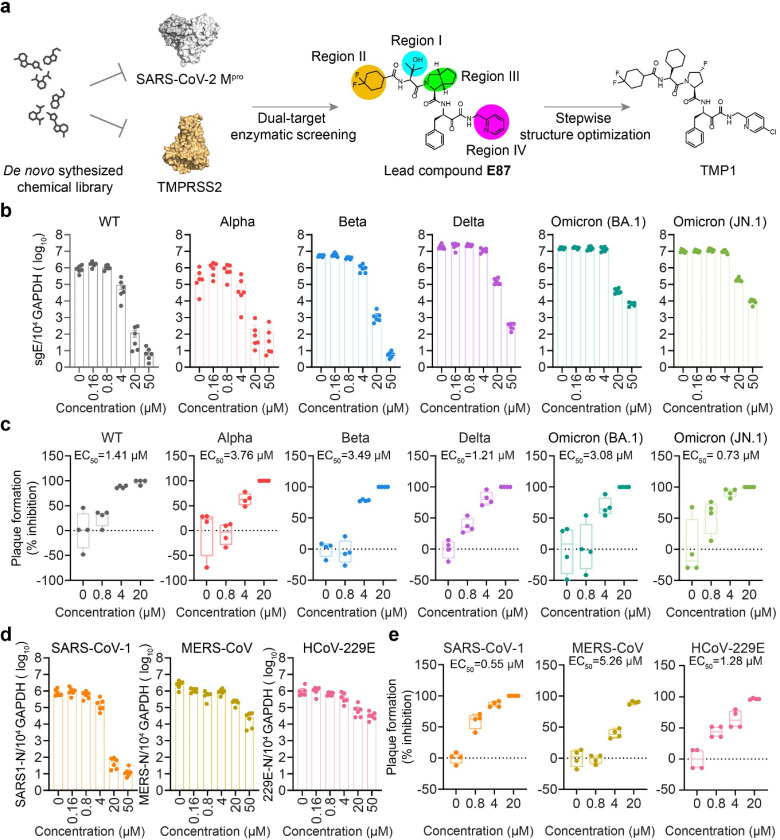
Discovery of the M^pro^/TMPRSS2 bispecific inhibitor with highly potent anti-coronavirus efficacy. (a) Schematic illustration of the screening workflow for the discovery of M^pro^/TMPRSS2 bispecific inhibitor. (b) Quantification of the subgenomic envelope (sgE) gene in VeroE6-TMPRSS2 cells (*n*=6) infected with wildtype SARS-CoV-2 and Alpha, Beta, Delta, Omicron (BA.1 and JN.1) variants in the presence or absence of TMP1. Lysates were harvested at 24 hpi. for one-step reverse transcription and quantitative polymerase chain reaction (RT-qPCR) analysis. (c) Infectious viral titres in the supernatants harvested at 24 hpi. from VeroE6-TMPRSS2 cells (*n*=4) infected with wildtype SARS-CoV-2 and Alpha, Beta, Delta, Omicron (BA.1 and JN.1) variants were determined by plaque assays. Number of plaques were normalized to those recovered from supernatants with mock treatment only. (d) Quantification of the nucleocapsid (N) gene in VeroE6-TMPRSS2 cells (*n*=6) infected with SARS-CoV-1 and MERS-CoV or in Huh7 cells infected with HCoV-229E at 24 hpi.. (e) Infectious viral titres in the supernatants harvested at 24 hpi. from VeroE6-TMPRSS2 cells (*n*=4) infected with SARS-CoV-1 and MERS-CoV or Huh7 infected with HCoV-229E were determined in VeroE6-TMPRRS2 (for SARS-CoV-1 and MERS-CoV) or Huh7 cells (for HCoV-229E) by plaque assays. Each data point represents one biological repeat. Data represents mean ± SD from the indicated number of biological repeats. Data were obtained from two or three independent experiments. WT, wildtype SARS-CoV-2.

To obtain a compound candidate with bispecific inhibition potential, we first established transfer (FRET)-based enzymatic assays with recombinant SARS-CoV-2 Omicron main protease (M^pro^) and the enzymatically-active TMPRSS2 ectodomain as we and others previously established^33,35^. We then screened an in-house *de novo* synthesized chemical library containing over 5000 compounds, resulting in a lead compound **E87** with both anti-M^pro^ and TMPRSS2 activity, exhibiting IC_50_ values of 368.6 nM against M^pro^ and 15.21 μM against TMPRSS2. (Fig. 1a and Table 1). We next proceeded to optimize its potency against both M^pro^ and TMPRSS2. The structural optimization was focused on Region-I to Region-IV on **E87** (Fig. 1a), and a step-wise optimization strategy was applied.

In the first step, we optimized Region-I with Region- , , and fixed as in **E87**. 8 new compounds (**10a-h)** with different fragments at Region-I were designed and synthesized. Bioactivities of these compounds are displayed in Table 1. Compound **10a** with the same fragment at Region-I (R^1^) as that of E87 but with a different configuration (R-configuration) showed decreased activity against both M^pro^ and TMPRSS2, indicating the S configuration was preferred at R^1^. We thus prepared a series of new compounds (**10b-g)** containing different substituents with S-configuration at R^1^. Compared with **E87**, compounds **10c-d** and **10f-h** showed increased activity against M^pro^, but only **10g** exhibited increased activity against TMPRSS2. The R^1^ substituent in **10g**, (S)-2-cyclohexyl, was thus set as an optimal fragment in the following optimization.

In the second step, we optimized Region-II with Region- , fixed as original subgroups, and Region- as the optimal (S)-2-cyclohexyl. 8 new compounds (**13a-h**) were designed and synthesized. Bioactivities of compounds **13a-h** are displayed in Table 2. All of the tested compounds showed significantly decreased activity against TMPRSS2 (> 30 μM), although some of them displayed increased activity against M^pro^. Therefore, Region II remained unchanged as that in **E87**.

In the third step, we optimized Region- with Region- and II as their optimal substituents and Region-IV as in **E87**. 10 new compounds (**18a-j**) were designed and synthesized. Bioactivities of compounds **18a-j** are displayed in Table 3. Compound **18a** generated by replacing (1S,3aR,6aS)-octahydrocyclopenta[c]pyrrole-1-formamide group with phenylalanine exhibited improved M^pro^ inhibitory activity with an IC_50_ value of 19.1 nM, while it completely lost activity against TMPRSS2, suggesting Region III specifically affects the inhibition against TMPRSS2. To explore the optimal inhibition against TMPRSS2, we prepared **18b-j**, containing different dicyclic or monocyclic groups in Region III. **18c-d** and **18f** showed increased activity against M^pro^, but decreased activity against TMPRSS2. Compounds **18e** and **18g-j** displayed increased activity against TMPRSS2, but decreased activity against M^pro^. To balance the inhibition against TMPRSS2 and M^pro^, we prioritized **18e** as the optimal compound in this step.

Finally, we optimized Region-IV with other regions fixed as their optimal fragments. We designed and synthesized four new compounds (**24a-d**). Bioactivities of these compounds are displayed in Table 4. All four compounds showed comparable potency against M^pro^ as **18e** but only **24d (TMP1)** displayed increased activity against TMPRSS2. Overall, through the above structural optimization and structure-activity relationship (SAR) studies, we obtained a series of α-ketoamide-containing dual inhibitors against M^pro^ and TMPRSS2. Among them, compound TMP1 is the most potent one with an IC_50_ value of 1.28 μM against TMPRSS2 and 312.5 nM against M^pro^.

Next, we comprehensively characterized the antiviral potency of TMP1 against wildtype SARS-CoV-2 and other variants of concern (VOCs) including Alpha, Beta, Delta and Omicron (BA.1 and JN.1) in VeroE6-TMPRSS2 cells. To exclude cellular cytotoxicity caused by TMP1 treatment, we performed in vitro cytotoxicity assays. Our results indicated no significant in vitro cytotoxicity was found at the therapeutic concentrations used in this study (Supplementary Fig. 1). Our results showed that TMP1 potently reduced both viral burden and infectious progeny viral titres in a dose-dependent manner in vitro (Fig. 1b and 1c). Plaque assays demonstrated comparable EC_50_ of TMP1 against wildtype SARS-CoV-2 and the tested VOCs, which varied between 0.73 to 3.76 μM (Fig. 1c). Given that M^pro^ is structurally conserved among the *Coronaviradae* family^29^ and that TMPRSS2 is a key host protease crucial for the entry of not only SARS-CoV-2 but also other human-pathogenic coronaviruses including the highly-pathogenic SARS-CoV-1 and MERS-CoV^3,12,13^, we examined the efficacy of TMP1 against other human-pathogenic coronaviruses. Our data suggested that TMP1 cross-protected against highly-pathogenic coronaviruses SARS-CoV-1 and MERS-CoV as well as the seasonal human coronavirus HCoV-229E in a dose-dependent manner (Fig. 1d), resulting in EC_50_ ranging from 0.55 to 5.26 μM (Fig. 1e). Collectively, our in vitro data demonstrates the novel M^pro^/TMPRSS2 bispecific inhibitor TMP1 not only suppresses the infection of SARS-CoV-2 but also potently protects against other seasonal and highly-pathogenic coronaviruses.

### Bispecific inhibitor TMP1 demonstrates potent antiviral efficacy in vivo and rescues hACE2 transgenic mice from lethal SARS-CoV-2 infection

**Figure 2:**
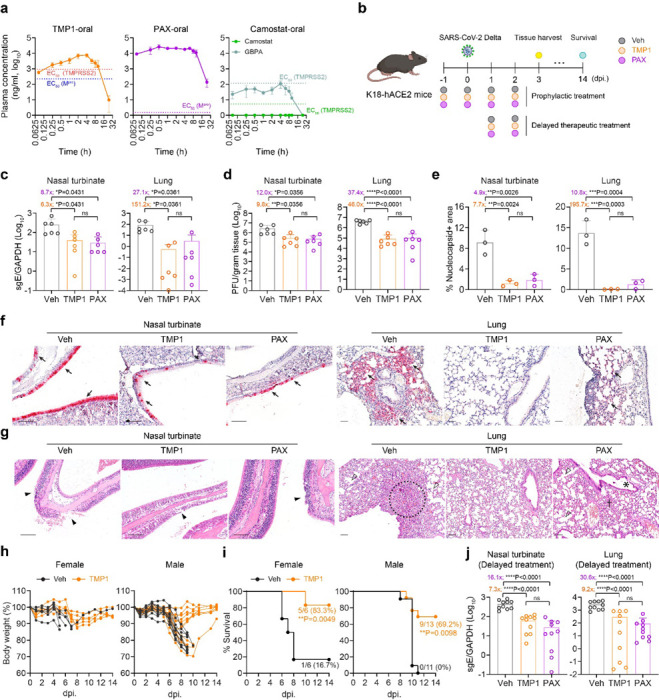
The in vivo antiviral efficacy of prophylactic and therapeutic TMP1 treatment against SARS-CoV-2 infection. (a) Pharmacokinetics of TMP1 oral delivery in mice. 8-week-old male BALB/c mice (*n*=3) were orally delivered with 100 mg/kg/dose TMP1, 100 mg/kg/dose nirmatrelvir (NRV) or 20 mg/kg/dose camostat. 20 mg/kg/dose ritonavir (RTV) was also included as metabolic enhancer for combined treatment. Plasma was continuously sampled for measurement of the plasma drug (or drug metabolites) concentration with liquid chromatography-mass spectrometry (LC-MS). (b) Schematic illustration of the in vivo experiment design. 8- to 12-week-old K18-hACE2 transgenic mice were intranasally challenged with 1250 PFU SARS-CoV-2 Delta strain. Mice were orally treated with 100 mg/kg/dose TMP1 or nirmatrelvir in combination with 20 mg/kg/dose ritonavir twice per day. For prophylactic therapy (*n*=6), treatment onset one day prior to virus infection while therapeutic treatment (*n*=10) was delayed to 24 hpi. Nasal turbinate and lung tissues were harvested at 3 dpi. for virological assessment by RT-qPCR and plaque assays. For survival study, body weight and survival of the infected mice were monitored for 14 days or until death of the animal. (c) Quantification of sgE gene of SARS-CoV-2 in the nasal turbinate and lung tissues of the infected mice with prophylactic treatment at 3 dpi by RT-qPCR analysis. (d) Quantification of the infectious viral titres in the nasal turbinate and lung tissues of the infected mice with prophylactic treatment at 3 dpi by plaque assays. (e) Viral antigen expression in the nasal turbinate and lung tissues of infected mice (*n*=3) with prophylactic treatment at 3 dpi. was quantified with ImageJ. (f) Representative images of SARS-CoV-2 nucleocapsid (N) protein expression (black arrow) in nasal turbinate and lung tissue of the infected mice at 3 dpi. by IHC staining. Scale bar represents 100 μm. (g) Histology analysis of the nasal turbinate and lung tissue of the infected at 3dpi. by H&E staining. Scale bar represents 100 μm. Black arrowhead, nasal epithelial desquamation; open arrowhead, alveolar collapse; dashed circle, inflammation infiltrations in alveolar septa; asterisk, bronchiolar epithelium damage. (h) Body weight change of the female (*n*=6) and male (*n*=11–13) infected mice with or without TMP1 prophylactic treatment. (i) Survival of the female (*n*=6) and male (*n*=11–13) infected mice with or without TMP1 prophylactic treatment. (j) Quantification of sgE gene of SARS-CoV-2 in the nasal turbinate and lung tissues of the infected mice with delayed therapeutic treatment at 3 dpi by RT-qPCR analysis. Each data point represents one biological repeat. Data represents mean ± SD from the indicated number of biological repeats. Statistical significances were determined using one way-ANOVA with Dunnett’s multiple comparisons test (c-e), (j) and log-rank (Mantel-Cox) tests (i). Data were obtained from three independent experiments. * represented p < 0.05 and ** represented p < 0.01. *** represented p < 0.001, **** represented p < 0.0001. ns, not statistically significant; WT, wildtype SARS-CoV-2; Veh, vehicle; PAX, Paxlovid.

Oral bioavailability of antiviral treatment is critical to its timely application during emerging pandemics. Therefore, we characterized the oral pharmacokinetics (PK) of TMP1 in Balb/c mice before in vivo antiviral efficacy evaluation. Our data suggested that upon single-dose oral administration (100 mg/kg TMP1 and 20 mg/kg ritonavir as metabolic enhancer), plasma concentration of absorbed TMP1 maintained above the EC_50_ against SARS-CoV-2 M^pro^ and TMPRSS2 for over 16 and 12 hours, respectively (Fig. 2a). The maximum blood concentration (C_max_) of 8028.86 μg/L (equivalent to 11.2 μM) was reached at 3.33 h post-delivery, resulting in an oral availability of 77.5% in mice (Table 5). The oral bioavailability of TMP1 in dogs was 132% (Table 5). To evaluate the redistribution of TMP1 in different organ tissues after absorption into the plasma, we measured the TMP1 concentration in lung, brain, liver, kidney and intestine tissues of the treated mice. Our results indicated that TMP1 could be maintained at 11.1-, 11.4- and 6.5-fold higher than its in vitro EC_50_ of SARS-CoV-2 Delta at 1, 2 and 8 hours post oral delivery (Supplementary Fig. 2). To exclude in vivo toxicity caused by TMP1, mice were given 150 mg/kg/dose, twice per day of TMP1 for a consecutive of 4 days. Body weight, kidney and liver function and tissue sections for histopathological analysis of the treated mice were monitored and we found no signs of in vivo toxicity at the given dosage and dosing frequency (Supplementary Fig. 3). In parallel, we examined the oral bioavailability of Paxlovid and camostat mesylate, which are clinically-approved M^pro^ and TMPRSS2 inhibitors for COVID-19 treatment^32,36^ (Fig. 2a). Consistent with previous results from the literature, plasma concentration of Paxlovid quickly reached over 10 μg/ml upon oral delivery^32^. To our surprise, no detectable level of camostat mesylate, but only its metabolite, 4-(4-guanidinobenzoyloxy) phenylacetic acid (GBPA), was found in the plasma after oral delivery. However, plasma concentration of GBPA remained below its EC_50_ against TMPRSS2 (Fig. 2a and Supplementary Fig. 4). Our data suggested that despite its strong anti-TMPRSS2 potency in vitro, oral delivery of camostat mesylate might not be an optimal therapeutic option for SARS-CoV-2 treatment in vivo. Consequently, we selected Paxlovid monotherapy over combined camostat mesylate and Paxlovid treatment for side-by-side comparison with TMP1 in subsequent in vivo assays.

We next proceeded to investigate the in vivo antiviral potency of TMP1 using the K18 human ACE2 (K18-hACE2) transgenic mice, which is a well-established model for COVID-19 research^37–39^. Briefly, mice were orally treated twice per day with vehicle only, TMP1 or Paxlovid. Treatment began at 1 day prior to challenge with SARS-CoV-2 Delta variant and lasted until day 2 post virus challenge (Fig. 2b). Viral genome quantification and infectious viral progenies titration at 3 days post infection (dpi.) showed that TMP1 significantly suppressed SARS-CoV-2 infection to a comparable level as Paxlovid in the nasal turbinates (Fig. 2c and 2d). Notably, viral burdens in the lung tissues were reduced to even lower levels in the TMP1-treated mice when compared with those treated with Paxlovid (reduction in lung viral gene copies: [TMP1 vs Paxlovid]: 151.2-fold vs 27.1-fold); reduction in lung infectious viral titres: [TMP1 vs Paxlovid]: 46.0-fold vs 37.4-fold) (Fig. 2c and 2d), though the differences have not yet reached statistical significance.

To examine viral antigen expression in vivo, we detected the coronavirus nucleocapsid (N) protein by immunohistochemistry (IHC) staining using specific anti-sarbecovirus N antibodies. Abundant amount of viral N protein was found in both nasal turbinate and lung tissues of vehicle-treated mice (Fig. 2e and 2f). In comparison, expression of N protein was significantly lowered by TMP1 treatment, which was further verified with quantification of the viral antigen positive area (Fig. 2e and 2f). Additionally, H&E staining was performed to identify infection-related histopathological lesions in the nasal turbinate and lung tissues. In line with the earlier virological findings, signs of virus-induced pathologies including loss of epithelium integrity, septal inflammation, alveoli deformation, and submucosal inflammatory infiltrations were found most evident in the vehicle-treated mice. On the contrary, these histopathological changes were largely alleviated or absent in their TMP1-treated counterparts (Fig. 2g).

Next, we sought to answer whether TMP1 treatment might rescue animals from lethal SARS-CoV-2 challenge. We infected the transgenic mice with lethal dose (1250 PFU per mouse) of SARS-CoV-2 Delta and monitored animal survival for 14 days. Vehicle-treated mice developed continuous body weight loss and began to succumb to lethal virus challenge as early as 6 dpi., resulting in 16.7% survival for the female mice and 0% for the male mice at 14 dpi. (Fig. 2h and 2i). In contrast, TMP1-treated mice were observed with significantly delayed onset of death (Vehicle: 6 dpi. vs TMP1: 9 dpi.) and the survival rate was significantly rescued to 83.3% (P = 0.0049) and 69.2% (P = 0.0098) for female and male mice, respectively (Fig. 2h and 2i). To explore the therapeutic potential of TMP1, we initiated the antiviral treatment at an delayed timepoint at 24 hpi.. We found that TMP1 still significantly lowered viral gene copies by 7.3- (P < 0.0001) and 9.2-fold (P < 0.0001) in the nasal turbinate and lung tissues, respectively (Fig. 2j). Together, our in vivo data suggests that prophylactic and therapeutic TMP1 treatments robustly reduce viral burdens in the infected mouse airways and ameliorate infection-associated tissue pathology, thus improving the overall survival of the infected animals.

### Bispecific inhibitor TMP1 substantially reduces SARS-CoV-2 infection in human airway epithelium and blocks SARS-CoV-2 transmission in golden Syrian hamsters

**Figure 3:**
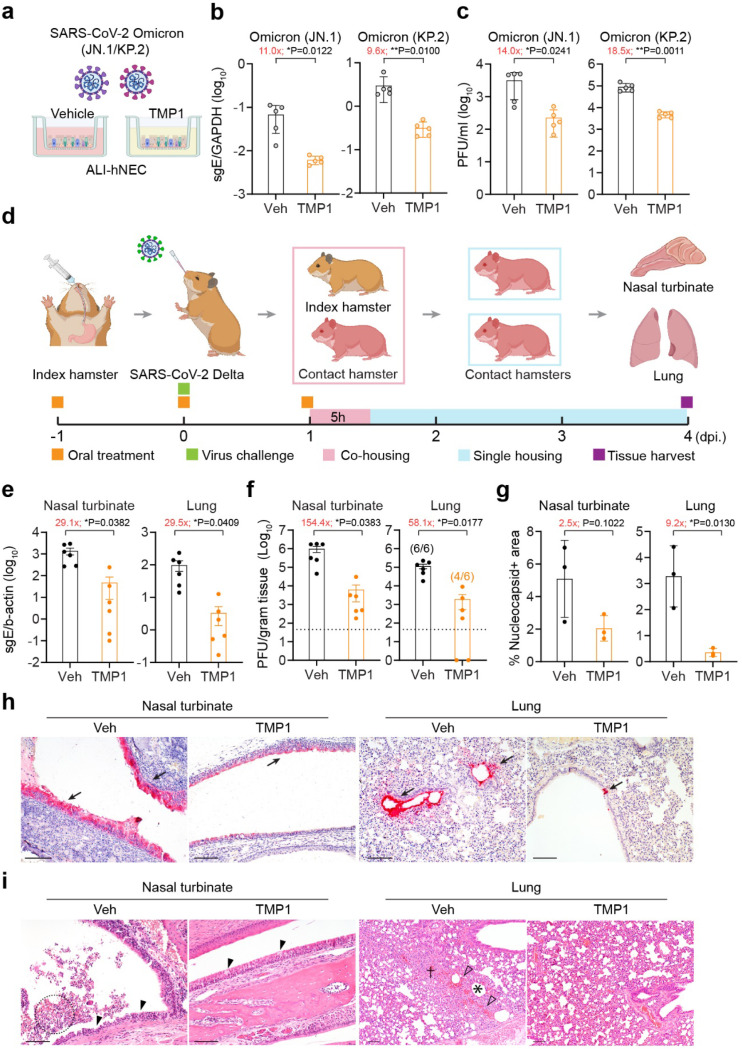
Efficacy of TMP1 in blocking SARS-CoV-2 transmission. (a) Schematic illustration of SARS-CoV-2 infection in human nasal epithelial cells (hNECs). Differentiated hNECs maintained in air-liquid interface (ALI) culture were pretreated with 20 μM TMP1 or vehicle for 1 hour. After 1 h, cells were washed and infected with SARS-CoV-2 Omicron JN.1 (*n*=5) or KP.2 (*n*=5). At 2 hpi., medium at the both apical and basal side were replenished with TMP1 or vehicle only until sample harvest at 48 hpi. (b) Quantification of sgE gene in the infected cell lysates at 48 hpi. by RT-qPCR analysis. (c) Quantification of the infectious viral titres in the apical supernatants harvested from the infected hNECs at 48 hpi. by plaque assays. (d) Schematic illustration of the transmission study in golden Syrian hamsters. Index hamsters (*n*=6) were orally treated with 90 mg/kg TMP1 together with 12 mg/kg RTV oral delivery of TMP1 or vehicle one day prior to infection. On the infection day (Day 0), index hamsters were infected with 2000 PFU SARS-CoV-2 Delta. Treatment in the index hamsters continued until they were co-housed with naïve contact hamsters (*n*=6) for 5 hours to allow virus transmission. Contact hamsters were separated for single housing until sample harvest on 4 dpi.. (e) Quantification of sgE gene of SARS-CoV-2 in the nasal turbinate and lung tissues of the contact hamsters at 4 dpi by RT-qPCR analysis. (f) Quantification of the infectious viral titres in the nasal turbinate and lung tissues of the contact hamsters 4 dpi by plaque assays. (g) Quantification of viral antigen expression in nasal turbinate and lung tissues of the contact hamsters at 4 dpi. by IHC staining. Quantification was performed with ImageJ. (h) Representative images of SARS-CoV-2 nucleocapsid (N) protein expression (black arrow) in nasal turbinate and lung tissue of the contact hamsters at 4 dpi. by IHC staining. Scale bar represents 100 μm. (i) Histology analysis of the nasal turbinate and lung tissue of the infected at 4 dpi. by H&E staining. Black arrowhead, nasal epithelial desquamation; dashed circle, necrotic cell debris in the nasal cavity; open arrowhead, haemorrhage in the alveolar septa; asterisk, alveoli collapse; cross, inflammatory infiltration in alveolar septa. Scale bar represents 100 μm. Each data point represents one biological repeat. Data represents mean ± SD from the indicated number of biological repeats. Statistical significances were determined using two-tailed Student’s *t*-test (b-c) and (e-g). Data were obtained from three independent experiments. * represented p < 0.05 and ** represented p < 0.01. Veh, vehicle.

TMPRSS2 expression in the airway epithelium is a key determinant of coronavirus transmission in vivo^24^. We next asked whether the improved bispecific design to include TMPRSS2 as one of the antiviral targets might help to block coronavirus transmission. To evaluate this question, we first infected the air-liquid interface-cultured human nasal epithelial cells (ALI-hNECs) with two SARS-CoV-2 Omicron prevalent subvariants JN.1 and KP.2 to mimic coronavirus infection at the primary infection site (Fig. 3a). Our results indicated that TMP1 treatment significantly decreased the viral gene copies by approximately one log and the infectious viral titres by more than one log for both subvariants in the infected ALI-hNECs (Fig. 3b and 3c), demonstrating the potential of TMP1 in suppressing SARS-CoV-2 infection in the human upper airways.

Next, we evaluated the impact of TMP1 treatment on SARS-CoV-2 transmission using the golden Syrian hamster transmission model as we and others previously established^40,41^. To better simulate the scenario in the human population, only the index but not the contact hamsters were treated. Briefly, index hamsters with or without TMP1 oral treatment were intranasally inoculated with 2000 PFU SARS-CoV-2 Delta and rested overnight. On the next day, the infected index hamsters were co-housed with naïve contacts for five hours to allow virus transmission. Contacts were then separated and individually housed for three more days until tissue harvest at 4 dpi. (Fig. 3d). Compared with contact hamsters in the control group, those in contact with the TMP1-treated hamsters experienced 29.1-fold (P = 0.0382) and 154.4-fold (P = 0.0383) lower viral gene copies and infectious viral titres in the nasal turbinates, respectively (Fig. 3e and 3f). Similarly, viral burdens in the lung tissues of contact hamsters of the TMP1 treatment group were remarkably lower (viral gene copies: 29.5-fold reduction, P = 0.0409; infectious viral titres: 58.1-fold reduction, P = 0.0177) than their control group littermates (Fig. 3e and 3f). Importantly, infectious progeny viruses were not recovered from two contact hamster lungs out of the six samples (33.3%) in the TMP1 contact group (Fig. 3f). In corroboration with viral burdens quantification, viral N expression was scarcely detected in the nasal turbinates or lungs of the TMP1 contact hamsters with IHC staining, which was dramatically different from the abundant viral N protein expression in the control contact hamsters (Fig. 3g and 3h). Histopathological analysis revealed that while extensive loss of integrity in the nasal epithelium and large amounts of necrotic cell debris in the nasal cavity were found in the control contact hamsters, only dispersed epithelial cell loss was occasionally detected in the TMP1 group (Fig. 3i). Consistently, while massive inflammatory infiltrations in alveolar septa and alveoli deformation were present in the control contact hamster lungs, these pathological findings were largely absent in the TMP1 contact hamsters (Fig. 3i). Together, our results demonstrate that our bispecific inhibitor TMP1 is effective against the prevalent Omicron strains in the human upper airway epithelium and effectively attenuates SARS-CoV-2 transmission in vivo.

### Bispecific inhibitor TMP1 cross-protects against highly-pathogenic human coronaviruses in vivo

**Figure 4:**
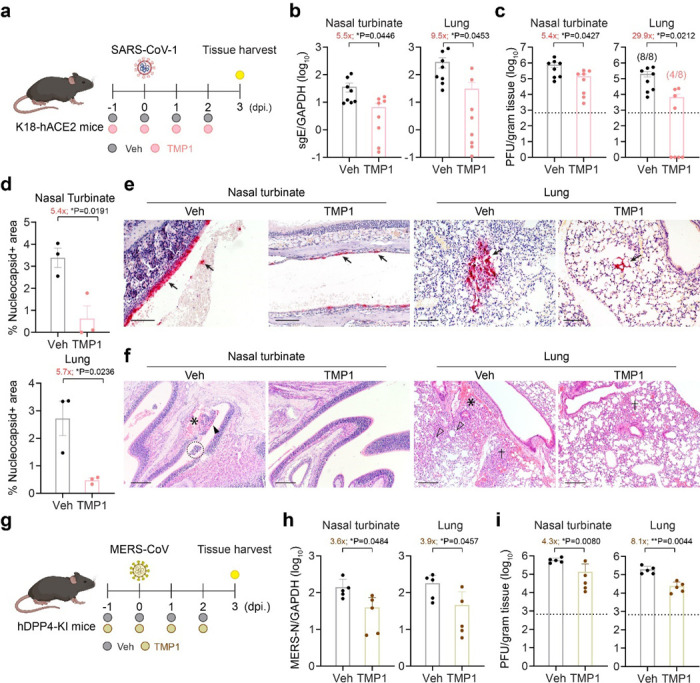
Cross-protection of TMP1 against highly-pathogenic human coronaviruses in vivo. (a) Schematic illustration of SARS-CoV-1 infection in K18-hACE2 transgenic mice. 8- to 12-week-old K18-hACE2 transgenic mice were intranasally infected with 500 PFU SARS-CoV-1. One day prior to infection, mice were orally treated with 100 mg/kg/dose TMP1 in combination with 20 mg/kg/dose RTV (*n*=8). Control mice were treated with vehicle only (*n*=8). Mice were treated twice per day until sample harvest at 3 dpi. (b) Quantification of sgE gene of SARS-CoV-1 in the nasal turbinate and lung tissues of the infected mice with prophylactic treatment at 3 dpi by RT-qPCR analysis. (c) Quantification of the infectious viral titres in the nasal turbinate and lung tissues of the SARS-CoV-1-infected mice at 3 dpi by plaque assays. (d) Viral antigen expression in the nasal turbinate and lung tissues of the SARS-CoV-1-infected mice (*n*=3) at 3 dpi. was quantified with ImageJ. (e) Representative images of SARS-CoV-1 nucleocapsid (N) protein expression (black arrow) in nasal turbinate and lung tissues of the SARS-CoV-1-infected mice at 3 dpi. by IHC staining. Scale bar represents 100 μm. (f) Histology analysis of the nasal turbinate and lung tissues of the SARS-CoV-1-infected mice at 3 dpi. by H&E staining. Black arrowhead, nasal epithelial desquamation; asterisk, haemorrhage in nasal submucosal region; dashed circle, necrotic cell debris in nasal cavity; open arrowhead, alveolar collapse; cross, inflammatory infiltration. Scale bar represents 200 μm. (g) Schematic illustration of MERS-CoV infection in hDPP4-knockin (hDPP4-KI) transgenic mice. 10- to 14-week-old hDPP4-KI mice were intranasally infected with 5000 PFU of mouse-adapted MERS-CoV. One day prior to infection, mice were orally treated with 100 mg/kg/dose TMP1 in combination with 20 mg/kg/dose RTV (*n*=5). Control mice were treated with vehicle only (*n*=5). Mice were treated twice per day until sample harvest at 3 dpi. (h) Quantification of N gene of MERS-CoV in the nasal turbinate and lung tissues of the infected mice at 3 dpi by RT-qPCR analysis. (i) Quantification of the infectious viral titres in the nasal turbinate and lung tissues of the MERS-CoV-infected mice at 3 dpi by plaque assays. Each data point represents one biological repeat. Data represents mean ± SD from the indicated number of biological repeats. Statistical significances were determined using two-tailed Student’s *t*-test (b-d) and (h-i). Data were obtained from three independent experiments. * represented p < 0.05 and ** represented p < 0.01. Veh, vehicle.

Inspired by the strong in vivo antiviral potency of TMP1 against SARS-CoV-2, we were interested in exploring the cross-protection of TMP1 against other highly-pathogenic human coronaviruses in vivo. To this end, we first examined the antiviral potency of TMP1 against SARS-CoV-1, which is also a human-pathogenic sarbecovirus that utilized ACE2 as entry receptor (Fig. 4a). Our results demonstrated that TMP1-treated SARS-CoV-1-infected K18-hACE2 transgenic mice experienced significantly lower viral burdens in both nasal turbinate and lung tissues when compared with that of the control mice (Fig. 4b). In particular, titres of infectious viral progenies were reduced by 29.9-fold in the TMP1-treated mouse lungs (Fig. 4c). Strikingly, four out of eight TMP1-treated transgenic mice developed no detectable level of infectious viral titres in the lungs, suggesting TMP1 treatment led to sterile protection in 50% of the challenged animals. In parallel, IHC staining also verified the viral load quantification and revealed that SARS-CoV-1 nucleocapsid protein expression was significantly reduced by 5.4-fold (P = 0.0191) and 5.7-fold (P = 0.0236) by TMP1 treatment in nasal turbinate and lung tissues, respectively (Fig. 4d and 4e). Concordantly, while histopathological findings including loss of epithelium integrity in the nasal mucosa and alveoli damage were evident in the control mice (Fig. 4f), the epithelial lining remained largely intact in the nasal turbinate of the TMP1-treated mice and only mild inflammatory infiltrations were discernible in the alveolar septa in the lungs (Fig. 4f).

To further extend our findings to other human-pathogenic coronaviruses that are more evolutionarily distant, we challenged human dipeptidyl peptidase 4 knock-in mice (hDPP4-KI mice) with a lethal dose (5000 PFU) of mouse-adapted MERS-CoV (MERS-CoV_MA_) with or without TMP1 oral treatment and harvested nasal turbinate and lung samples on 3 dpi. for virological assessments (Fig. 4g). In keeping with our earlier findings with SARS-CoV-1, TMP1 treatment suppressed MERS-CoV_MA_ replication in both nasal turbinates and lungs when compared with control mice (Fig. 4h). Infectious viral titres were decreased by 3.6- (P = 0.0219) and 8.1-fold (P = 0.0044) in the nasal turbinate and lung tissues, respectively (Fig. 4i). Consistent with the viral burden findings, expression of MERS-CoV N protein was reduced in TMP1-treated mouse nasal turbinate and lung tissues (Supplementary Fig. 5a). Additionally, histological analysis indicated TMP1 treatment alleviated virus-induced epithelial damage in the nasal turbinate and reduced alveolar destruction in the infected mice (Supplementary Fig. 5b). Together, these in vivo findings with SARS-CoV-1 and MERS-CoV infection corroboratively supports our hypothesis that oral administration of the M^pro^/TMPRSS2 bispecific inhibitor confers broad-spectrum antiviral protection against highly-pathogenic coronaviruses.

### Bispecific inhibitor TMP1 binds to TMPRSS2 enzymatic pocket to suppress TMPRSS2-dependent coronavirus entry

**Figure 5:**
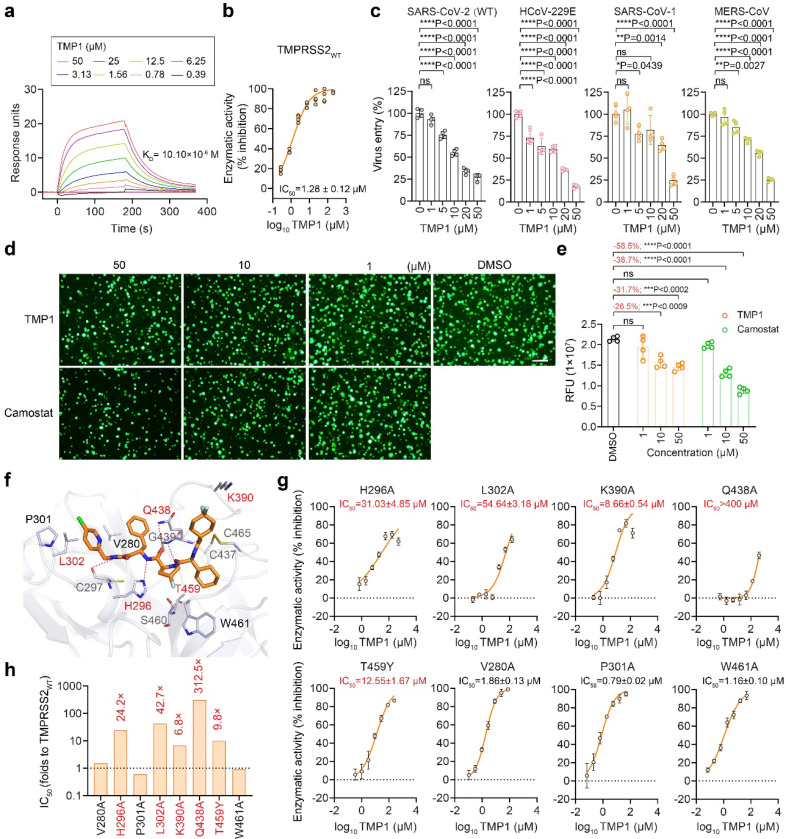
Specific inhibition of TMP1 against TMPRSS2 enzymatic activity and TMPRSS2-dependent pseudovirus entry. (a) Surface plasmon resonance (SPR) analysis of TMP1 with TMPRSS2. (b) Enzymatic activity of recombinant TMPRSS2 with TMP1 treatment. Enzymatic activity of the recombinant TMPRSS2 was measured by fluorescence resonance energy transfer (FRET) assays (*n*=4). Fluorescence signals were normalized to the readouts of mock-treated wells. (c) Inhibition of pseudovirus entry by TMP1. VeroE6-TMPRSS2 and Huh7 cells were pre-treated with TMP1 for 1 h. VeroE6-TMPRRS2 cells were transduced with pseudoviruses carrying SARS-CoV-2 wildtype spike (S) (*n*=4). Huh7 cells transfected with TMPRSS2 were transduced with pseudoviruses carrying SARS-CoV-1-S (*n*=4), MERS-CoV-S (*n*=4) or HCoV-229E-S (*n*=4). Pseudovirus entry was quantified by measuring the luciferase signal at 24 hours post transduction. Luminescence signals were normalized to the readouts of mock-treated wells. (d) Representative images of TMPRSS2-dependent cell-cell fusion. 293T cells were co-transfected with SARS-CoV-2-S and GFP1–10 (effectors cells). Target cells followed were co-transfected with hACE2, TMPRSS2, and GFP11 (target cells). Prior to effector and target cell co-culture, target cells were pre-treated with TMP1 or camostat for 30 mins, followed by co-culture at 1:1 ratio for 24 hours in the presence of TMP1 and camostat. TMPRSS2-mediated cell-cell fusion was visualized by immunofluorescence microscope. Scale bar represents 200 μm. (e) Quantification of the fluorescence signals of cell-cell fusion assays as described in Figure 5d. Quantification of the fluorescence signals were performed with ImageJ. RFU, relative fluorescence units. (f) Mode of binding between TMPRSS2 (in blue-white, PDB accession: 7MEQ) and TMP1 (in orange). Residues in close proximity of the interaction interface were shown as blue-white sticks. Key amino acids confirmed by mutagenesis assays were highlighted in red. The distally-located amino acid W461 included as negative control in the mutagenesis assay was also shown. Hydrogen bonds were represented as red dashed lines. (g) Enzymatic assays with TMPRSS2 mutants carrying key residues located in the TMP1-TMPRSS2 interaction interface. Enzymatic activities of the recombinant TMPRSS2 mutants with or without TMP1 treatment were determined by FRET-based enzymatic assays (*n*=4). Enzymatic activities were determined by normalization of the fluorescence signals to the readouts of mock-treated control wells. (h) Fold change of change in IC_50_ of TMP1 against TMPRSS2 mutants compared with wildtype TMPRSS2. Each data point represents one biological repeat. Data represents mean ± SD from the indicated number of biological repeats. Statistical significances were determined using one way-ANOVA with Dunnett’s multiple comparisons test (c) and (e). Data were obtained from three independent experiments. * represented p < 0.05 and ** represented p < 0.01, **** represented p < 0.0001, ns, not statistically significant.

To mechanistically demonstrate that TMP1 indeed possesses specific inhibition against the targeted host protease TMPRSS2, we set out to characterize its binding with TMPRSS2 by surface plasmon resonance (SPR) analysis. The SPR analysis revealed direct interaction between TMP1 and TMPRSS2 with a *K*_D_ value of 10.10 × 10^− 6^ M (Fig. 5a). FRET-based enzymatic assay further confirmed TMP1 inhibited the protease activity of recombinant TMPRSS2 at an EC_50_ of 1.28 μM (Fig. 5b). TMPRSS2 cleaves the spike proteins at S2’, exposing the fusion peptide to facilitate virus entry^42^. To study the anti-TMPRSS2 potency of TMP1 independent of its anti-M^pro^ activity, we first investigated whether TMP1 might reduce TMPRSS2-mediated coronavirus entry. To this end, we pre-treated VeroE6-TMPRSS2 and Huh7 cells with TMP1 followed by infection of SARS-CoV-2 spike-pseudoviruses. Measurement of luciferase signals indicated that TMP1 dose-dependently reduced SARS-CoV-2 pseudovirus entry (Fig. 5c). Similarly, TMP1 prevented the entry of HCoV-229E, SARS-CoV-1 and MERS-CoV pseudoviruses, showing a broad spectrum of inhibition on TMPRSS2-dependent coronavirus entry (Fig. 5c). Additionally, we performed the split GFP assay that found TMP1 efficiently suppressed TMPRSS2-dependent cell-cell fusion mediated by SARS-CoV-2 wildtype spike in side-by-side comparison with camostat mesylate (Fig. 5d and 5e). To exclude the possibility of off-target inhibition against other host proteases, we cross-examined the potential inhibition of TMP1 against a panel of other host proteases including calpain1, cathepsin L/D and thrombin (Table 6). Our data indicated that TMP1 was highly selective against TMPRSS2 over the other human cysteine/serine proteases.

To illustrate the binding mode of TMP1 with TMPRSS2, we tried to solve the co-crystal structure of TMPRSS2 in complex with TMP1 but it was unsuccessful. Alternatively, we performed molecular docking to predict possible interactions between TMP1 and TMPRSS2, followed by verification with mutagenesis assays. As shown by the molecular docking analysis, TMP1 resided in a large hydrophobic pocket of TMPRSS2, which contained a catalytic site formed by the catalytic triad of H296, D345, and S441 (Fig. 5f). It formed strong hydrophobic interactions with P301, L302, V280, K390, H296, and C465. Additionally, TMP1 formed five hydrogen bonds (H-bonds) with residues Q438, G439, H296, and C297 (Fig. 5f).

To verify the interface as predicted by our docking model, we mutated seven residues (V280, H296, P301, K390, Q438, L302, and T459) in TMPRSS2 that were predicted to be in close proximity with TMP1 (Fig. 5f). The distantly-located W461 was included as a negative control. Substitutions of Q438, L302, H296, and K390 to alanine resulted in a significant reduction in TMP1 potency by 312.5, 42.7, 24.2, and 6.8-fold, respectively (Fig. 5g and 5h), indicating their crucial roles in TMP1 binding to TMPRSS2. Moreover, T459Y substitution, which introduced a larger side chain and causes steric hindrance (Supplementary Fig. 6) substantially reduced TMP1 potency by 9.8-fold (Fig. 5g and 5h). As expected, mutation W461A did not significantly affect the potency of TMP1 against TMPRSS2 (Fig. 5g and 5h). Together, these results of strongly support the molecular docking model, demonstrating that TMP1 occupies the catalytic pocket of TMPRSS2 to inhibit its enzymatic activity and prevent TMPRSS2-mediated coronavirus entry.

### Bispecific inhibitor TMP1 suppresses SARS-CoV-2 M^pro^ through covalent binding and inhibits nirmatrelvir-resistant SARS-CoV-2 infection in vivo

**Figure 6:**
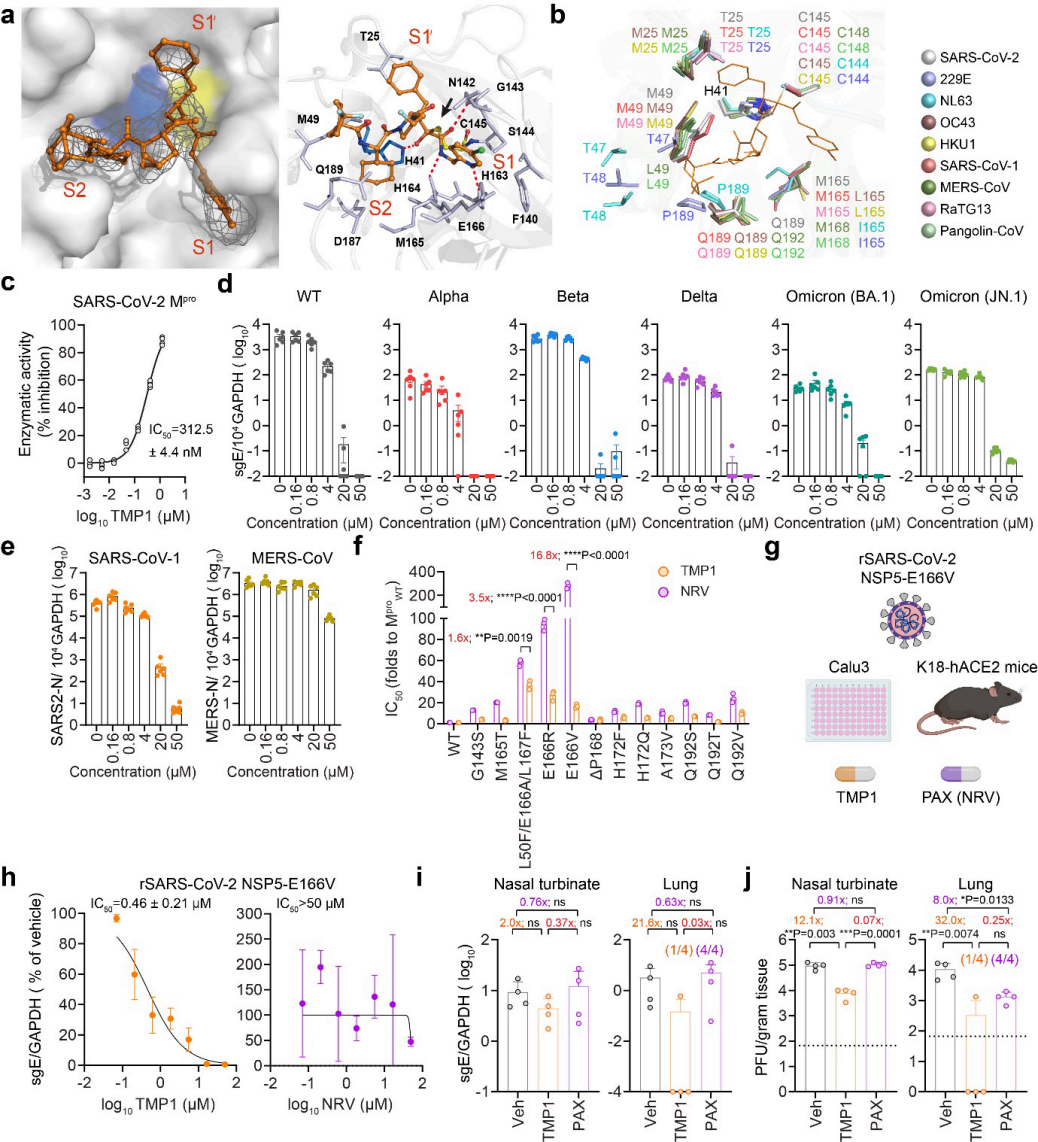
Specific inhibition of TMP1 against coronavirus M^pro^ and its antiviral efficacy against nirmatrelvir-resistant SARS-CoV-2 escape mutant. (a) Crystal structure of TMP1 in complex with SARS-CoV-2 Omicron M^pro^. Left panel, The co-crystal structure (PDB: 9IZB) of TMP1 (orange) in complex with SARS-CoV-2 Omicron M^pro^ (grey). The H41 (blue) and C145 (yellow) catalytic dyad was shown. The S1′, S1, and S2 pockets of M^pro^ are labelled in red. The *Fo-Fc* electron density map of TMP1 is shown in gray mesh (σ = 2.5). Right panel, close-up view of TMP1 with the substrate binding pocket of M^pro^. The residues of M^pro^ involved in TMP1 binding were displayed by sticks. The hydrogen bonds were displayed as red dashed lines. The covalent-bond between Cys145 and TMP1 warhead was indicated by a black arrow. (b) Superimposition of the TMP1 in complex with M^pro^ from 9 coronaviruses including SARS-CoV-2 (Omicron, PDB: 9IZB), HCoV-229E (PDB: 2ZU2), -NL63 (7E6M), -OC43, -HKU1, SARS-CoV-1 (PDB: 1WOF), MERS-CoV (PDB: 4RSP), RaTG13 and GX/P3B. (c) Enzymatic activity of recombinant SARS-CoV-2 M^pro^ with TMP1 treatment. Enzymatic activity of the recombinant SARS-CoV-2 M^pro^ was measured by fluorescence resonance energy transfer (FRET) assays (*n*=4). Fluorescence signals were normalized to the readouts of mock-treated wells. (d) Quantification of the sgE gene in VeroE6 cells (*n*=6) infected with wildtype SARS-CoV-2 and Alpha, Beta, Delta, Omicron (BA.1 and XBB1.5) variants, followed by treatment with TMP1 or vehicle only at 1 hpi.. Lysates were harvested at 24 hpi. for one-step reverse transcription and quantitative polymerase chain reaction (RT-qPCR) analysis. (e) Quantification of the N gene of SARS-CoV-1 and MERS-CoV in VeroE6 cells (*n*=6) infected with SARS-CoV-1 or MERS-CoV, followed by treatment with TMP1 or vehicle only at 1 hpi.. Lysates were harvested at 24 hpi. for one-step reverse transcription and quantitative polymerase chain reaction (RT-qPCR) analysis. (f) Sensitivity of recombinant SARS-CoV-2 M^pro^ mutants to TMP1 treatment. Inhibition of TMP1 against the recombinant SARS-CoV-2 M^pro^ mutants carrying reported nirmatrelvir-resistant mutations was measured by fluorescence resonance energy transfer (FRET) enzymatic assays (*n*=3). Fold change in the IC_50_ was obtained by comparing with that of the wildtype M^pro^. (g) Schematic illustration of characterizing the in vitro and in vivo antiviral efficacy of TMP1 against nirmatrelvir-resistant recombinant SARS-CoV-2. Recombinant SARS-CoV-2 was constructed with NSP5-E166V mutation in the background of ancestral SARS-CoV-2 with D614G mutation in the spike (rSARS-CoV-2-NSP5-E166V). For in vitro infection, Calu3 cells were pretreated with TMP1 for 1 hour followed by infection with rSARS-CoV-2-NSP5-E166V (*n*=4). Lysates were harvested at 24 hpi. for RNA extraction. For in vivo infection, 8- to 12-week-old K18-hACE2 transgenic mice were challenged with 5000 PFU rSARS-CoV-2-NSP5-E166V. One day prior to infection, mice were orally treated with 100 mg/kg/dose TMP1 in combination with 20 mg/kg/dose RTV (*n*=4). Control mice were treated with vehicle only (*n*=4). Mice were treated twice per day until sample harvest at 3 dpi. (h) Quantification of the sgE gene in Calu3 cells (*n*=6) infected with rSARS-CoV-2-NSP5-E166V, followed by treatment with TMP1 or vehicle only at 1 hpi.. Lysates were harvested at 24 hpi. for one-step reverse transcription and quantitative polymerase chain reaction (RT-qPCR) analysis. (i) Quantification of SARS-CoV-2 sgE gene in the nasal turbinate and lung tissues of the rSARS-CoV-2-NSP5-E166V infected mice at 3 dpi by RT-qPCR analysis. (j) Quantification of the infectious viral titres in the nasal turbinate and lung tissues of the rSARS-CoV-2-NSP5-E166V infected mice at 3 dpi by plaque assays. Each data point represents one biological repeat. Data represents mean ± SD from the indicated number of biological repeats. Statistical significances were determined using one way-ANOVA with Dunnett’s multiple comparisons test (i-j) and two-tailed Student’s *t*-test (f). Data were obtained from three independent experiments. * represented p < 0.05, ** represented p < 0.01, *** represented p < 0.001, ns, not statistically significant. Veh, vehicle; NRV, nirmatrelvir; PAX, Paxlovid.

To better understand the mode of inhibition of TMP1 against M^pro^, we determined the crystal structure of the M^pro^-TMP1 complex at approximately 2.6 Å (PDB ID: 9IZB). The electron density map illustrated the binding mode of TMP1 with M^pro^ (Fig. 6a, **left panel**). The 5-chloropyridine at P1′ inserted into the S1 pocket, while the P1 benzyl group occupied S1′. The (R)-4-fluoropyrrolidine at P2 pointed towards the solvent region, and the (S)-2-cyclohexyl at P3, along with the 4,4-difluorocyclohexyl at P4, inserted into S2. The carbonyl carbon of TMP1’s α-ketoamide warhead formed a reversible covalent bond (~ 1.8 Å) with C145’s Sγ atom in the (R)-configuration (Fig. 6a, **right panel**). The hydroxy group of this thiohemiketal formed a hydrogen bond with H41 (~ 2.4 Å). The amide oxygen of TMP1 formed a hydrogen bond with G143 (~ 2.7 Å) and was oriented toward the “oxyanion hole” formed by the backbones of G143 and C145. The P1′ group formed hydrogen bonds with H163 (~ 3.2 Å) and H164 (~ 2.5 Å), and also engaged in hydrophobic interactions with F140, N142, M165, and E166. The P2 moiety was solvent-exposed, indicating flexibility for various functional groups. The P3 and P4 groups inserted into the hydrophobic S2 pocket, interacting with M49, D187, and Q189 (Fig. 6a, **right panel**). Simulation of TMP1 in complex with the M^pro^ of HCoV-229E, HCoV-NL63, HCoV-OC43, HCoV-HKU1, SARS-CoV-1, MERS-CoV, RaTG13 (bat-CoV) and GX/P3B (pangolin-CoV), supported the broad-spectrum inhibition potential of TMP1 against human-pathogenic coronaviruses and other mammalian sarbecoviruses (Fig. 6b and Supplementary Fig. 7). FRET-based enzymatic assay indicated a dose-dependent inhibition of TMP1 against recombinant SARS-CoV-2 M^pro^, resulting in an IC_50_ of 312.5 nM (Fig. 6c).

To study the antiviral potency of TMP1 independent of its anti-TMPRSS2 activity, we challenged the TMPRSS-2-deficient VeroE6 cells with wildtype SARS-CoV-2 and different VOCs. TMP1 treatment was started at the post-entry stage at 1 hour post infection (hpi.). Our data showed that the post-entry TMP1 treatment was still capable of lowering viral gene copies in SARS-CoV-2-infected VeroE6 cells (Fig. 6d), supporting that the bispecific inhibitor TMP1 indeed suppressed coronavirus replication by targeting coronavirus M^pro^. In addition, we also investigated the possibility of broad-spectrum inhibition of TMP1 against the M^pro^ of other human-pathogenic coronaviruses. Our results suggested that TMP1 similarly decreased SARS-CoV-1 and MERS-CoV replication in VeroE6 cells in a dose-dependent manner (Fig. 6e), indicating that TMP1 not only inhibited the activity of SARS-CoV-2 M^pro^ but also that of the two highly-pathogenic coronaviruses.

Paxlovid has been most widely used as oral antiviral for COVID-19 treatment. Nevertheless, emerging nirmatrelvir (NRV)-resistant SARS-CoV-2 mutants has become a major public health concern^43–45^. Crystal structure data obtained in this study suggested the mode of interaction between TMP1 and M^pro^ were different from that of NRV (Fig. 6a and Supplementary Fig. 8). We further demonstrated with FRET assays that TMP1 was 16.8-fold less sensitive to NSP5-E166V mutation when compared with NRV (Fig. 6f), which is a dominant mutation leading to NRV-resistance found in COVID-19 patients^45,46^. The bispecific antiviral design is advantageous in combating drug-resistance caused by mono-target therapeutics. Therefore, we were interested to explore whether TMP1 might protect against the infection of NRV-resistant SARS-CoV-2 variants. To address this research question, we constructed recombinant SARS-CoV-2 (rSARS-CoV-2) that carried the NSP5-E166V mutation in the background of ancestral SARS-CoV-2 with D614G mutation in the spike (Fig. 6g). We subsequently infected Calu3 cells with rSARS-CoV-2 NSP5-E166V, followed by TMP1 or NRV treatment. Expectedly, NRV treatment was ineffective against the recombinant virus (IC_50_ > 50 μM) (Fig. 6h). In sharp contrast, TMP1 dose-dependently decreased the intracellular viral gene copies of rSARS-CoV-2 NSP5-E166V in Calu3, resulting in an IC_50_ of 0.46 μM (Fig. 6h). We next asked whether antiviral efficacy of TMP1 against the NRV-resistant rSARS-CoV-2 was preserved in hACE2 transgenic mice. In concordance with the in vitro data, viral gene copies of rSARS-CoV-2 NSP5-E166V were reduced by TMP1 treatment in the nasal turbinate and lung tissues of the infected mice (Fig. 6i). Remarkably, infectious viral titres were decreased by 12.1- (P = 0.003) and 32.0-fold (P = 0.0074) by TMP1 treatment in the nasal turbinate and lung tissues, respectively (Fig. 6j). In comparison, infectious viral titres in the Paxlovid-treated mice nasal turbinates were 14-fold (P = 0.0001) higher than that of the TMP1-treated mice (Fig. 6j). In keeping with the infectious viral titres, only scarcely distributed viral antigen was found in the nasal turbinate of the TMP1-treated mice, while that of the vehicle- or Paxlovid-treated mice were extensively detected along the nasal epithelium (Supplementary Fig. 9). Similarly, TMP1 treatment also reduced the N protein expression in the infected lung tissue samples when compared with the vehicle or Paxlovid treatment group (Supplementary Fig. 9). Histological analysis further supported that the reduced viral burdens in the TMP1-treated mouse nasal turbinate and lung tissues led to alleviated virus-induced pathological changes (Supplementary Fig. 9). Together, our mechanistic data indicates that TMP1 interacts with M^pro^ using a distinct mechanism when compared with NRV, therefore retaining the sensitivity against nirmatrelvir-resistant SARS-CoV-2 escape mutants in vitro and in vivo.

In summary, our orally-available bispecific inhibitor TMP1 simultaneously suppresses the activity of SARS-CoV-2 M^pro^ and host TMPRSS2 with high potency, thus effectively blocking the infections and transmission of human-pathogenic coronaviruses.

## Discussion

The continuous emergence of SARS-CoV-2 variants have imposed great challenges to antiviral therapy development. Monotherapy targeting a single viral protein is often associated with rapid emergence of escape mutations as we have seen in the clinical application of remdesivir^47,48^ and Paxlovid^43,46,49^. Here, we described the discovery of a bispecific inhibitor TMP1 which simultaneously targeted the viral protease M^pro^ and the host protease TMPRSS2. We demonstrated the potent antiviral efficacy of TMP1 against wildtype SARS-CoV-2 and other variants of concern, including the prevalent Omicron JN.1 and KP.2 subvariants (Fig. 1 and Fig. 3). With side-by-side comparison with Paxlovid, we found both prophylactic and therapeutic treatment with TMP1 were comparably efficacious in protecting hACE2 transgenic mice from SARS-CoV-2 infection, thus ameliorating virus-induced tissue pathologies and lethality (Fig. 2). Interestingly, TMP1 not only inhibited the infection of SARS-CoV-2 but also protected against other human-pathogenic coronaviruses including the highly pathogenic SARS-CoV-1 and MERS-CoV in vivo (Fig. 4).

Priming of the spike protein by host proteases prior to fusion either at the plasma membrane or endosomes is essential for coronavirus entry. Therefore, a wealth of antiviral studies has targeted important host proteases such as TMPRSS2, cathepsin B/L and calpain^18,50–52^. Although coronaviruses can use a variety of host proteases to facilitate virus entry in cells with low or little TMPRSS2 expression, TMPRSS2-mediated membrane fusion remains as the dominant entry pathway utilized by coronaviruses for efficient virus entry in the human airway^3,12–15,22,23,25,26,53,54^. Therefore, we reasoned that targeting TMPRSS2 is physiologically relevant to the design of anti-coronavirus inhibitors. To this end, we performed stepwise chemical modifications to further optimized the anti-TMPRSS2 activity of our bispecific inhibitor (Fig. 1 and Table 1 to 4). To demonstrate the specific inhibition against TMPRSS2 in the context of coronavirus infection, we showed that TMP1 dose-dependently reduced the entry of pseudoviruses expressing spikes from a panel of human-pathogenic coronaviruses (Fig. 5) whose entry were previously shown to be TMPRSS2-dependent^3,12–14^. In parallel, TMP1 also inhibited TMPRSS-mediated cell-cell fusion (Fig. 5), which contributes to coronavirus spread and pathogenicity in vivo^25,26^.

Despite the efforts in developing TMPRSS2 inhibitors, therapeutic options against TMPRSS2 as antivirals for coronavirus infections remained limited^3,18,19,55^. The most well-studied TMPRSS2 inhibitor camostat mesylate were used to treat COVID-19 patients^3,56^, yet has led to inconclusive outcomes^36,57–61^. Although camostat mesylate potently suppresses the TMPRSS2 enzymatic activity with single-digit nanomolar IC_50_ values in biochemical assays, its rapid in vivo turnover into the metabolites with significantly diminished effect against TMPRSS2 substantially compromised its antiviral potency in clinical trials^11,57^. We measured the PK properties of camostat in mice with an oral dosage equivalent to that was used in COVID-19 patients^36,61^ and found the amount of camostat and GBPA in the plasma fell below their effective concentrations (Fig. 2 and Supplementary Fig. 3). In comparison, the high oral bioavailability of TMP1 conferred effective inhibition against both TMPRSS2 and M^pro^ for over 12 hours (Fig. 2), thus allowing feasible antiviral therapy for patients in outpatient clinics and at home.

As the first FDA-approved oral drug for COVID-19 treatment, Paxlovid has been widely used since first launched into the market^32^. However, its strong antiviral potency might be undermined by the emergence of NRV-resistant mutations^43,62,63^. NRV is a peptidomimetic inhibitor which acts by covalent binding with the enzymatic pocket of M^pro^. In vitro virus passaging and recombinant virus experiments demonstrated T21I, L50F, E166A/V, S114A, ΔP168, A173V/T in the M^pro^ contributed to NRV resistance^44,49,62–64^. Alarmingly, among the clinical isolates identified with NRV resistance, E166V was found as the second most frequently-detected mutation in the M^pro^, which contributed to 17.9% of NRV-resistant mutations^45,64^. In line with reports from others^43,49^, we found that E166V most significantly reduced the sensitivity of SARS-CoV-2 M^pro^ to NRV (more than 200-fold increase in EC_50_) (Fig. 6). Therefore, the need of developing alternative therapeutic options in combating NRV-resistance is warranted. Hinted by our crystallization analysis that TMP1 interacted with M^pro^ in a distinctive mode when compared to NRV, we verified that TMP1 remained highly potent against the M^pro^ carrying NRV-resistant mutations. Furthermore, TMP1 suppressed the replication of NRV-resistant recombinant SARS-CoV-2 in human lung cell lines and in transgenic mice (Fig. 6).

Combination therapy with antivirals targeting distinct virus or host proteins essential to the virus life cycle are invaluable that they enhance the treatment potency by synergistic inhibition and also reduce the emergence of drug-resistant mutant viruses, as exemplified by the highly active antiretroviral therapy (HAART) against human immunodeficiency virus (HIV) infection^65^. Synergistic anti-SARS-CoV-2 efficacy of the combination treatment with the orally-available Paxlovid and Molnupiravir was recently reported in rhesus macaques^66^, yet the potential benefits of combination treatment in humans needs to be further verified with randomized clinical trials. On the other hand, recent studies on the discovery of single antiviral molecules possessing dual-inhibition mechanisms further advanced the coronavirus antiviral research^50,51,67–74^. Since coronavirus M^pro^ and the host cathepsin L are both cysteine proteases that share structural similarity in the enzymatic pocket, majority of the dual-target inhibitors were discovered from screening of known M^pro^ inhibitors against the anti-cathepsin L activities^50,67–69,73^. However, given the indispensable role of TMPRSS2 in coronavirus entry in the human airways, we are the first study to adopt a novel approach to simultaneously target coronavirus M^pro^ and TMPRSS2 by the bispecific inhibitor TMP1. Among the reported anti-coronavirus inhibitors with dual-target, in vivo antiviral potency was only available for four small molecules (GC376, Olgotrelvir, SMI141 and SMI142)^51,73,75^. In comparison with these M^pro^/cathepsin L-targeting inhibitors, TMP1 showed improved animal survival against lethal coronavirus challenge, alleviated pathological changes in the infected tissues and strong capacity in blocking coronavirus transmission, therefore verifying the physiological importance of the antiviral targets selected in our current design.

In summary, we developed an orally-available bispecific inhibitor TMP1 which simultaneously targets coronavirus M^pro^ and the host TMPRSS2. Virological assessment demonstrated the potent anti-coronavirus efficacy of TMP1 in vitro. Besides, TMP1 significantly ameliorated virus-induced lung pathology and rescued infected animals from lethal coronavirus infection, supporting its potential in preventing severe infection in patients which requires hospitalization. Overall, our research provides a proof of concept that simultaneously targeting the coronavirus protease M^pro^ and the key host protease TMPRSS2 by a dual-inhibitor represents a promising strategy of anti-coronavirus therapy development.

## Methods

### Cell lines

Huh7, VeroE6 and 293T cells obtained from ATCC were maintained in Dulbecco’s Modified Eagle medium (DMEM) supplemented with 10% heat-inactivated fetal bovine serum (FBS), 100 U/ml penicillin, and 100 μg/ml streptomycin (1% P/S). Calu3 cells obtained from ATCC were maintained in DMEM/F12 supplemented with 10% FBS, 1% P/S. VeroE6-TMPRSS2 cells were obtained from the Japanese Collection of Research Bioresources (JCRB) Cell Bank and cultured also in 10% FBS, 1% P/S DMEM and 1mM (1%) sodium pyruvate.

### Virus and safety

WT SARS-CoV-2 HKU-001a (GenBank: MT230904), B.1.617.2/Delta (GenBank: OM212471), B.1.1.529/Omicron BA.1 (GenBank: OM212472), JN.1 (GISAID: EPL_ISL_18841631) and KP.2 (GISAID: EPI_ISL_19351035) were isolated from patients with laboratory confirmed COVID-19 in Hong Kong^76^. MERS-CoV (GenBank: JX869059.2) was a gift from R. Fouchier (Erasmus Medical Center, Rotterdam, The Netherlands). The mouse-adapted MERS-CoV_MA_ was a gift from P. McCray (University of Iowa, IA, USA). SARS-CoV GZ50 (GenBank: AY304495) and HCoV-229E (GenBank: PQ243243) were archived clinical isolate at the Department of Microbiology, The University of Hong Kong (HKU). All variants of SARS-CoV-2 were cultured and titrated by plaque assays using VeroE6-TMPRSS2 cells. MERS-CoV and SARS-CoV-1 were propagated and titrated by plaque assays in VeroE6 cells. HCoV-229E was propagated and titrated by plaque assays in Huh7 cells. After obtaining the virus culture, the sequences of all variants used in this study were confirmed with nanopore sequencing. In vivo and in vitro experiments concerning live SARS-CoV-1, SARS-CoV-2 and MERS-CoV were performed according to the approved standard operating procedures of the Biosafety Level 3 facility at Department of Microbiology, HKU.

### Chemical synthesis and inhibitors used in vivo

TMP1 and its derivatives were synthesized in-house as described in Table 1 to 4. Nirmatrelvir, camostat mesylate, 4-Hydroxy Benzeneacetic Acid 2-(Dimethylamino)-2-oxoethyl Ester (FOY-251) and ritonavir were purchased from MedChemExpress (USA, NJ, HY-138687, HY-13512, HY-19727A, HY-90001).

### Animals and ethics approval

Heterozygous K18-hACE2 C57BL/6J mice (2B6.Cg-Tg(K18-ACE2)2Prlmn/J) were obtained from The Jackson Laboratory. The hDPP4 exon 10 to 12 KI mice were provided by P. McCray (University of Iowa, IA, USA) and were previously described^77,78^. Golden Syrian hamsters were obtained from the Centre for comparative Medicine Research of the University of Hong Kong. The BALB/c mice were obtained from Sichuan University. The animals were kept in cages with individual ventilation under 65% humidity and an ambient temperature of 21–23°C and a 12–12 h day–night cycle for housing and husbandry. Food and water were provided to the animals without restriction. Group sizes were chosen based on statistical power analysis and our prior experience. Gender- and age-matched mice were randomized into different experimental groups. The use of animals was approved by the Committee on the Use of Live Animals in Teaching and Research of The University of Hong Kong and the Institute Animal Care and Use Committee (IACUC) of West China Hospital, Sichuan University.

### Characterization of the in vitro toxicity of TMP1

VeroE6-TMPRSS2, VeroE6 and Calu3 were treated with TMP1 diluted at the designated concentrations and incubated for 48 hpi.. Cell viability was measured with luminescence-based CellTiter-Glo luminescent cell viability assay kit (G7573, Promega, WI, USA), following manufacturer’s manual with the GloMax Explorer Multimode Microplate Reader (Promega). Luminescence signals are normalized with solvent controls.

### Evaluation of in vitro antiviral activity

Calu3, VeroE6-TMPRSS2, VeroE6 or Huh7 cells were infected with SARS-CoV-2 (WT, Alpha, Beta, Delta, Omicron BA.1 or JN.1), recombinant SARS-CoV-2 NSP5-E166V, MERS-CoV, or HCoV-229E at multiplicity of infection (MOI) ranging from 0.01 to 2. Unless specified, cells were pre-treated with serially-diluted TMP1, nirmatrelvir or vehicle only for 1 hour prior to virus infection. 2 μM CP-100356 was added as P-glycoprotein efflux inhibitor in antiviral assays conducted with VeroE6 and VeroE6-TMPRSS2 cells. Inhibitors were removed during virus infection by PBS washing for three times. After 2-hour incubation, the inoculum was removed and replaced with supernatants supplemented with inhibitors. Cells were incubated at 37°C until sample harvest.

### RNA extraction and one-step reverse transcription-quantitative polymerase chain reaction (RT-qPCR)

Viral RNA was extracted from infected cells using QIAsymphony RNA Kit (931636, Qiagen, Germany). Viral RNA from mice lung and nasal turbinate samples were extracted with the RNeasy Mini kit (74106, Qiagen). After RNA extraction, RT-qPCR was performed using QuantiNova Probe RT-PCR Kit (208354, Qiagen) or QuantiNova SYBR Green RT-PCR Kit (208154, Qiagen) with the LightCycler 480 Real-Time PCR System (Roche). The primers and probes used in this study was included as Supplementary Table 1.

### Virus titration with plaque assays

For organ harvested from infected animals, tissues were homogenized in DMEM with Tissue Lyzer II (Qiagen) and cleared supernatants are collected after centrifugation. To determine the infectious virus titre, supernatants from infected cells or organ tissues were ten-fold serially diluted and inoculated into monolayered VeroE6-TMPRSS2 cells (for quantification of SARS-CoV-1, SARS-CoV-2 and MERS-CoV) or Huh7 cells (for quantification of HCoV-229E) with 2h incubation at 37°C, followed by 1% low-melting point agarose overlay (16520050, Thermofisher, USA). The cells were further incubated for 48h or 72h before fixation with 4% formaldehyde for visualization with 0.5% crystal violet diluted in 25% ethanol/distilled water as previously described^79^.

### Air-liquid interface culture of primary human nasal epithelial cells (hNECs) and virus challenge in hNECs

The human nasal epithelial cells in air-liquid interface (ALI) culture were purchased from Epithelix (EP02MP, Epithelix, Switzerland) and maintained with MucilAir culture medium (EP04MM, Epithelix) until virus challenge. On the day of virus challenge, cells were pre-treated with or without 20 μM TMP1 for 2 hours, followed by virus inoculation at the apical side. Cells were incubated for 2 h at 37°C to allow virus entry. Residual inoculum was removed and replaced with medium with or without TMP1. Apical supernatants and cell lysates were harvested for viral genome copy quantification by one-step RT-qPCR and infectious virus titration with plaque assays at 48 hpi..

### Characterization of the toxicity of TMP1 in mice

Female 6- to 8-week-old K18-hACE2 transgenic mice were orally treated with 150mg/kg/dose TMP1 or solvent only twice per day from day 0 to day 3. Blood was drawn from mice 24 h post the last dose of treatment and analysed for the concentration of aspartate transaminase (AST), aspartate transaminase (ALT) and creatine (Cr) in the plasma according to the manufacturer’s instruction (BC1555, BC1565 and BC4915, Solarbio, China). Body weight of the treated mice was measured daily. Histological analysis of major organ tissues harvested on day 14 post treatment.

### Virus challenge with SARS-CoV-1, SARS-CoV-2 and MERS-CoV and drug treatment in mice

For SARS-CoV-2 infection, 8- to 12-week-old K18-hACE2 transgenic mice anaesthetized with 100 mg/kg ketamine and 10 mg/kg xylazine were intranasally inoculated with 1250 PFU SARS-CoV-2 Delta variant. For SARS-CoV-1 infection hACE2 transgenic mice were challenged with 500 PFU SARS-CoV-1. For MERS-CoV infection, hDPP4-KI mice were challenged with 5000 PFU mouse-adapted MERS-CoV. For drug treatment, hACE2 transgenic mice were orally treated with 100 mg/kg/dose TMP1 or nirmatrelvir in combination with 20 mg/kg/dose ritonavir or vehicle only twice per day. hDPP4-KI mice were orally treated with 150 mg/kg/dose TMP1 in combination with 20 mg/kg/dose ritonavir or vehicle only twice per day. For prophylactic therapy, treatment onset one day prior to virus infection while therapeutic treatment was delayed to 24 hpi.. Nasal turbinate and lung tissues were harvested at 3 dpi. for virological assessment by RT-qPCR and plaque assays. For survival study, body weight and survival of the infected mice will be monitored for 14 days or until death of the animal, whichever was earlier.

### Hamster transmission study

Transmission study was performed in golden Syrian hamsters as previously established^40,80^. 8- to 10-week-old female and male index hamsters were orally treated with 90 mg/kg/dose TMP1 in combination with 20 mg/kg/dose ritonavir twice on the day prior to virus challenge. At 0 dpi., hamsters were anaesthetized with standard 200mg/kg ketamine and 10 mg/kg xylazine, followed by intranasal inoculation with 2000 PFU SARS-CoV-2 Delta strain. The index hamsters were rested for 16 h before being co-housed with the naïve hamsters for 5 hours at 1 dpi. to allow contact transmission. Treatment for the index hamsters were continued twice per day until the end of virus transmission period at 1 dpi.. Contact hamsters were then returned to single housing until sample harvest at 3 days post exposure (4 dpi.) for virological and histological assessments.

### Immunohistochemistry (IHC) staining and histology analysis

IHC staining was performed to detect viral proteins from animal organ tissue samples. The nucleocapsid protein (NP) of MERS-CoV was detected by in-house guineapig polyclonal anti-MERS-CoV NP antibody and SARS-CoV-1 and SARS-CoV-2 was detected using in-house rabbit polyclonal anti-SARS-CoV NP antibody, followed by incubation with biotinylated rabbit anti-guineapig IgG (H + L) (ab6770, Abcam, UK) or biotinylated goat anti-rabbit IgG (H + L) secondary antibody (BA-1000, Vector laboratories, USA). Specificity of the inhouse primary antibody was validated as previously described^81,82^. The colour development was carried out with VECTASTAIN^®^ ABC-AP Kit and VectorRed substrate kit (AK-5000 and SK-5100, Vector Laboratories) according to the manufacturer’s instructions. The nuclei counterstaining was performed by Gill’s haematoxylin followed by mounting with Vectamount permanent mounting medium. For H&E staining, the tissue sections are stained with Gill’s haematoxylin (H-3401–500, Vector Laboratories) and eosin-Y. All images are acquired by Olympus BX53 light microscope. IHC quantification was performed with IHC Image Analysis Toolbox as previously reported^23^.

### Pseudovirus package and entry assays

SARS-CoV-2-wildtype spike, SARS-CoV-1-spike, MERS-CoV-spike and HCoV-229E-spike pseudoviruses were packaged as previously described^83,84^. Briefly, 293T cells were transfected with different spikes with Lipofectamine 3000 (L3000–015, Thermo Fisher Scientific). At 24 h post transfection, the cells were transduced with VSV-deltaG-firefly pseudotyped with VSV-G. At 2 h post transduction, the cells were washed three times with PBS and cultured in DMEM containing 1%FBS and anti-VSV-G (8G5F11) antibody (EB0010, Kerafast, MA, USA). The pseudoviruses were then harvested at 16 h post transduction and titrated with TCID_50_ assays.

For pseudovirus entry assays, DMSO-dissolved TMP1 was diluted in DMEM containing 2% FBS to the designated concentrations resulting in 0.5% DMSO in the working solutions. Infection of peudoviruses carrying SARS-CoV-2-S was performed in VeroE6 TMPRSS2 cells. Infection of peudoviruses carrying SARS-CoV-1-S, MERS-CoV-S and HCoV-229E-S was performed in Huh7 cells. Cells were pre-treated with TMP1 for 2 h, followed by pseudovirus infection for 2 h. The cells were then incubated in an incubator (37, 5% CO_2_) for 24 h, before washed and lysed for detection of luciferase signal with a luciferase assay system (DD1204, Vazyme, China) according to manufacturer’s instructions.

### Cell-cell fusion assay with camostat and TMP1 treatment

Cell-cell fusion assay was adapted from a protocol as we previously described^83,85^. Briefly, 293T cells were co-transfected with different SARS-CoV-2 wildtype spike plasmid with GFP1–10 plasmid (cat#68715, Addgene, USA) as effector cells. Another population of 293T cells was co-transfected with ACE2, TMPRSS2, and GFP11 (cat#68716, Addgene) as target cells. After 24 h post-transfection, the target cells were treated with serially-diluted TMP1 or camostat for 1 h. For control wells, cells were treated with 0.5% DMSO in DEM. Effector and target cells were subsequently digested by EDTA–Trypsin (25200–072, Gibco) and mixed at a 1:1 ratio. The mixed cells were co-cultured at a 37°C incubator for another 24 h. TMP1 and camostat were maintained in the supernatants during incubation. The co-cultured cells were fixed in 10% formalin and then permeabilized with 0.1% Triton-X100 (Sigma, USA) at room temperature. The antifade mounting medium with 4′,6-Diamidino-2-Phenylindole, Dihydrochloride (DAPI, H-1200, Vector Laboratories) was used for mounting and DAPI staining. Images were taken with the Olympus BX73 fluorescence microscope (Olympus Life Science, Tokyo, Japan).

### Molecular docking of TMP1 against TMPRSS2 and M^pro^

Molecular docking was implemented in the GOLD module with the GoldScore fitness function^86^ using slow search settings. For docking of TMP1 with TMPRSS2, the receptor structure was taken from the protein data bank (PDB) (PDB entry: 7MEQ) and pre-processed including filling in missing sidechains, removing waters, adding hydrogen atoms. Flexible docking was performed by defining the area within 10Å around the 7MEQ ligand as binding site, and the amino acid sidechains of the binding site were set as flexible. All other parameters were set to default values.

For docking of TMP1 with coronavirus M^pro^s, the protein structures of eight coronavirus main proteases were taken from PDB^87^ including SARS-CoV-1 (PDB entry: 1WOF), MERS-CoV (PDB entry: 4RSP), NL63 (PDB entry: 7E6M) and 229E (PDB entry: 2ZU2), or from the AlphaFold Protein Structure Database (https://alphafold.ebi.ac.uk) including OC43 (GenBank: YP_009555250.1), HKU-1 (GenBank: YP_173236.1), RaTG13 and GX/P3B. The genome sequences of RaTG13 (GISAID: EPI_ISL_402131) and GX/P3B (GISAID: EPI_ISL_410543) were downloaded from GISAID (https://www.gisaid.org/) and translated to protein sequences. Then, those structures were pre-processed by filling in missing sidechains, removing waters, adding hydrogen atoms, and aligned to the SARS-CoV-2 main protease structure (PDB entry: 9IZB). Flexible docking was performed by defining the area within 8Å around the 9IZB ligand (TMP1) as binding site, and the amino acid sidechains of the binding site were set as flexible. For each protein, the predicted pose of the ligand with the smallest root mean square error (RMSD) to TMP1 was selected as the binding pose. All other parameters were set to default values. The entire process of molecular docking was implemented in Discovery Studio 3.1.

### Design and cloning of TMPRSS2 and its mutation constructs

The gene coding human TMPRSS2 (GenBank: KJ897688.1) was synthesized by the GENERAL BIOL, Anhui, China. The PCR fragment including TMPRSS2 ectodomain (residues 109–492) was amplified and inserted into the pFastBac1vector using restriction sites EcoRI and *HindIII,* with a signal peptide GP64 (baculovirus envelope glycoprotein) at the N-terminus, and a 6×His tag at the C-terminus. The autoactivation sequence _250_SRQSR_255_↓IVGGE (the arrow indicates the cleavage site) of TMPRSS2 was replaced with an enterokinase-cleavage sequence _250_DDDDK_255_. The eight mutations of TMPRSS2 (V280A, H296A, P301A, L302A K390A, Q438A, T 459Y, and W461A) were constructed using single-point mutation method. Plasmid transfer vector containing the wild-type or mutant TMPRSS2 ectodomain gene was transformed into *E. Coli* DH10 Bac strain to generate recombinant viral Bacmid DNA. All primers are presented in Supplementary Table 2.

### Protein expression, purification, and activation of wild-type and mutant TMPRSS2 ectodomain

Taken the wild-type TMPRSS2 ectodomain as an example, Sf9 cells were transfected with Bacmid DNA using LipoInsect^™^ transfection reagents (C0551, Beyotime Biotechnology, China), according to the manufacturer’s instructions. After 96 h post-infection, P0 (about 2 mL) viral stock was collected and amplified to produce P1 to P3 (about 10 mL) viral stock. Two liters of sf9 cells cultured in the SIM SF Expression Medium (MSF1, Sino Biological, China) were infected with the P3 virus. TMPRSS2 ectodomain was then expressed and secreted outside cells at 27°C under shaking at 110 rpm within 4–5 d after baculovirus infection.

Next, the supernatant of cell culture was collected by centrifugation with 7500 × g, at 4°C, 10 min, to remove the cell pellet. Subsequently, the supernatant was incubated with 5ml Ni NTA with shaking at 110 rpm, 2 h, at 16°C, and transferred to a gravity flow column. The protein bound to the Ni-NTA column was washed using TBS buffer (25 mM Tris, pH 8.0, 150 mM NaCl), then was eluted using TBS buffer plus 250 mM imidazole. The eluted sample was concentrated to about 2 mg/mL and dialyzed to the reaction buffer (25 mM Tris, pH 8.0, 150 mM NaCl, 2 mM CaCl_2_). Enterokinase (C620031, Sangon Biotech., China) was added into the eluted sample, to activate the TMPRSS2 zymogen. Then, the C-terminal His-tag was removed using TEV protease. The sample was concentrated and loaded to the Superdex 75 gel filtration column in size-exclusion chromatography buffer (25 mM Tris, pH 8.0, 75 mM NaCl). Finally, the target protein was concentrated about 10 mg/mL and stored at −80°C.

### Fluorescence resonance energy transfer assay with recombinant M^pro^ and TMPRSS2

22.5μL optimized concentration of recombinant protease was pre-incubated with 2.5μL test compound in reaction buffer (M^pro^ and its mutants: 20 mM HEPES buffer, pH 6.5, 120 mM NaCl, 0.4 mM EDTA and 20% glycerol. TMPRSS2 and its mutants: 50mM Tris, pH 7.5, 250mM NaCl and 25% glycerol) at 96-well plates for 10min. Followed by the addition of 25 μL 20μM FRET substrate (M^pro^ and its mutants: MCA-AVLQSGFR-Lys (DNP) -Lys-NH2. TMPRSS2 and its mutants: Boc-QAR-AMC). The fluorescence signal was read at 320/405 nm (for M^pro^ and its mutants) and 360/460 nM (for TMPRSS2 and its mutants) on the CLARIOstar microplate reader (BMG).

### Surface plasmon resonance (SPR) analysis

SPR experiments were carried out using a Biacore 8K SPR system (GE Healthcare). Recombinant TMPRSS2 was immobilized on a Series S CM5 chip by amine coupling until the SPR signal reached ∼5,000 RU (resonance units) at a flow rate of 10 μL/min. Different concentrations of TMP1 were then passed through the CM5 chip through flow cells for 120 s followed by a 120-s dissociation phase at a flow rate of 30 μL/min. Background binding to blank immobilized flow cells was subtracted, and equilibrium dissociation constant (*K*_D_) values were calculated using the 1:1 binding kinetics model built in Biacore 8K Evaluation Software.

### In vivo pharmacokinetics of TMP1, Paxlovid and camostat

Male ICR mice (*n* = 3 per group) were treated by intravenous (i.v.) or by oral gavage (p.o.). The vehicle was consisted with 10% DMSO, 40% PEG400, 10% HS-15 and 40% saline. After administration, the blood samples (0.2 mL) were collected with 1 mL syringes containing anticoagulants (EDTA-2K and heparin) at indicated time points and analyzed by liquid chromatography tandem mass spectrometry (LC-MS/MS).

### The expression and purification of SARS-CoV-2 Omicron variant M^pro^ and its mutants

The method for expressing and purifying the Omicron variant (BA.5, GenBank: OP054053) M^pro^ and its mutants is consistent with our previously described procedures^33,88^. In brief, the cDNA sequence was cloned into the pET-28b vector, within the M^pro^ cleavage-site at the N-terminus and the PreScission cleavage-site at the C-terminus. The cloned plasmid was expressed in *E. coli* BL21(DE3) cells, and cultured in LB with Kanamycin (50 μg/ml) at 37°C. Upon reaching an optical density (600 nm) of 0.6–0.8, induction was carried out by adding 0.5 M IPTG (18°C, 18 h). Cell pellets were resuspended in the buffer (20 mM Hepes pH 7.5, 500 mM NaCl, 10 mM imidazole, 0.5 mM PMSF and 10% glycerol), followed by lysis via high-pressure homogenization. Subsequently, the lysate was clarified through centrifugation (18000 rpm for 45 min at 4°C), and the supernatant was loaded onto the His-Trap FF column (GE Healthcare) for purification. After the addition of PreScission protease to remove the His tag overnight, the protein was further purified using a Superdex 75 Increase 10/300 GL column (GE Healthcare).

### Crystallization, data collection, phase determination, and refinement

The purified M^pro^ (~ 5 mg/mL) and TMP1 were mixed at a molar ratio of about 1:10 and incubated on ice for 2 h. The mixture was then centrifuged at 13,000 rpm for 10 min. The sitting-drop vapor-diffusion technique was utilized, incorporating 1 μL of M^pro^-TMP1 mixture and 1 μL of the reservoir at a temperature of 291K. Crystals of M^pro^-TMP1 were observed under the condition: 0.2 M Magnesium chloride hexahydrate, 0.1 M Tris pH 8.5, 25% w/v PEG3350 at 18°C for 1 week. The crystal was fished out and flash-cooled in liquid nitrogen.

The X-ray diffraction experiment for M^pro^-TMP1 were collected at the BL18U1 beamline (wavelength = 0.97853 Å, temperature = 100 K) of the Shanghai Synchrotron Radiation Facility (SSRF). The obtained dataset was processed using *XDS*^89^ and scaled with Aimless in *CCP4*^90^. Molecular replacement was then performed on M^pro^-TMP1 structure using the M^pro^ (PDB ID: 7C7P) as an initial model. Model building was subsequently carried out using Coot and the refinement of the structure was performed using *PHENIX.refine*^91^. The final statistics of data collection and structural refinement are shown in Supplementary Table 3.

### Construction of recombinant SARS-CoV-2 with nirmatrelvir resistance

The cDNA from ancestral SARS-CoV-2 (strain HKU-001a), assembled into the pSMART-BAC vector by seamless assembly (E2621S, NEB, USA), was used as the background to generate a D614G amino acid substitution in the S gene and the and a E166V substitution at NSP5 gene, respectively. The mutations were introduced into the pSMART-BAC by site directed mutagenesis and confirmed by Sanger sequencing. The recombinant clones with mutant sites were transformed into BAC-Optimized Replicator v2.0 Electrocompetent Cells (60210–1, LGC Biosearch Technologies, UK), followed by plasmids extraction to acquire ultrapure and high quality of full-length cDNA clone. Infectious virus is recovered by transfection of VeroE6-TMPRS2 cells with 2.5 μg of the full-length cDNA clone using Lipofectamine 3000 (L3000015, Thermo Fisher Scientific). At 48 h post-transfection, the supernatant was used to inoculate VeroE6-TMPRSS2 cells for viral passage. The recombinant virus was sent to next generation sequencing to confirm the desired mutation and the absence of undesired mutations in the viral genome.

### Graphic illustration

Schematic illustration images were created with Adobe Illustrator CC2018 and BioRender software (https://biorender.com/).

### Statistical analysis

Statistical comparison between two experimental groups were performed with unpaired two-tailed Student’s t-test. Comparison between three or more experimental groups was performed with one-way ANOVA or two-way ANOVA. The survival of animals was compared using the log-rank (Mantel-Cox) test. 50% inhibitory concentration (IC_50_) and 50% effective concentration (EC_50_) were calculated by simple liner regression model and dose-response model in GraphPad Prism 8.0 software. Differences were considered statistically significant when p < 0.05. Data analysis was performed with GraphPad Prism v.8.0.

## Supplementary Material

Supplement 1

## Data Availability

Coordinates and maps associated with data reported in this manuscript was deposited to the d Protein Data Bank (PDB) with accession number 9IZB.
