# Supplementary material for: An orally available Mpro/TMPRSS2 bispecific inhibitor with potent anti-coronavirus efficacy in vivo
Source: Res Sq. 2024 Nov 21:rs.3.rs-5454588. Preprint. [Version 1] doi: 10.21203/rs.3.rs-5454588/v1 (PMC11601862; doi:10.21203/rs.3.rs-5454588/v1)
Supplement: Supplement 1 [file NIHPPRS5454588V1-supplement-1.pdf]

Supplementary Files

This is a list of supplementary files associated with this preprint. Click to download.

- 20241114SI.docx
- Tables.docx
